# Supplementary material for: Are carbon emissions trading and green financial instruments synergistic? -Comprehensive quantitative research based on content analysis
Source: PLoS One. 2024 Mar 7;19(3):e0298601. doi: 10.1371/journal.pone.0298601 (PMC10919601; doi:10.1371/journal.pone.0298601)
Supplement: S1 Data — (ZIP) [file pone.0298601.s001.zip › Data/地级市Z_F工作报告.zip!/║╙─╧/╓ú╓▌╩╨/└┤╫╘╣1⁄2╓┌║┼í╛┬φ┐╦╩2╛▌═°í┐_2004.docx]

各位代表：

　　现在，我代表市人民政府，向大会作工作报告，请予审议，并请各位政协委员提出意见。

　　一、过去五年的工作回顾

　　市第十一届人民代表大会第一次会议以来，全市人民在市委的领导下，坚持以邓小平理论和“三个代表”重要思想为指导，抓住机遇，开拓创新，加快发展，圆满完成了“九五”计划，提前实现了现代化建设的第二步战略目标，胜利完成了本届政府的各项任务。

　　(一)经济发展步入新阶段，综合实力迈上新台阶

　　五年来，我们始终坚持以经济建设为中心，克服世界经济波动，特别是东南亚金融危机带来的不利影响，国民经济持续快速健康发展，经济实力显著增强。全市生产总值年均增长11.3%，2003年首次突破千亿元大关，达到1102.1亿元，比上年增长14.7%，人均生产总值达到1917美元，标志着我市经济进入了新的发展阶段；地方财政收入由1998年的34.2亿元增加到72.5亿元，年均增长18.7%；金融机构年末存款余额由833.9亿元增加到2434.1亿元，年均增长23.9%；社会消费品零售总额由285.7亿元增加到479.9亿元，年均增长10.9%。城市综合竞争力有较大幅度提升。

　　(二)经济结构调整成效显著，农业、工业和商贸服务业协调发展

　　五年来，我们坚持不懈地进行经济结构的战略性调整，三次产业结构由6.3 53.7 40调整为4.4 52.1 43.5。

　　农业和农村经济结构逐步优化，畜牧养殖、林果、高效经济作物和优质专用农作物的比重进一步提高。无公害农产品基地和特色农产品示范区建设步伐加快。耕地总量保持动态平衡。五年来，全市农林牧渔业增加值由38.8亿元增加到48.8亿元；造林77.7万亩；农田水利基本建设和扶贫开发成效显著，先后解决32万人吃水问题，使10.5万人脱贫。

　　工业经济快速发展，优势产业规模日益扩大。积极调整工业经济结构和产业布局，以高新技术改造传统产业，着力培育大企业大集团，工业经济增长势头强劲，总量不断增加。2003年全市规模以上工业完成增加值297.4亿元，年均增长11.7%；规模以上工业企业年销售收入874.9亿元，年均增长12.3%；实现利税首次突破百亿，达到107.3亿元。煤电铝、汽车、食品等优势产业规模日益扩大，电子信息、生物工程与制药、新材料等新兴产业方兴未艾。年销售收入10亿元以上的企业由3户增加到11户。五年技改投入159.7亿元，各级企业技术中心由4户发展到43户。氧化铝、公路客车、速冻食品、纺织机械、耐火材料、阀门等产品在全国市场占有较大份额。三全、思念、金星啤酒3家企业的5种产品获得中国名牌。

　　第三产业蓬勃发展，商贸城建设步伐加快。批发零售、餐饮和房地产等传统服务业巩固壮大，物流、会展和旅游等现代服务业发展势头良好。新型业态发展迅速，连锁、超市、专业店由250家发到800多家，3家商业零售企业进入全国百强。商品交易市场辐射力增强，有10多家市场进入全国前列。易初莲花、麦当劳、肯德基以及丹尼斯、上海联华、北京华联等一大批国内外知名商业企业抢滩郑州。二七广场改造竣工，德化商业步行街投入使用，二七商圈购物环境明显改善。房地产业蓬勃发展，五年累计开发商品房1420万平方米。会展经济在探索中不断壮大，多次成功举办了郑州全国商品交易会和糖酒交易会。物流业建设取得进展，联邦等世界四大快递公司以及香江、东方等物流企业入驻郑州。假日经济屡创新高。旅游业快速发展，年旅游总收入由45.2亿元增加到104.9亿元，年均增长18.3%。

　　(三)投资力度不断加大，经济发展后劲明显增强

　　实施投资拉动战略，一批重点项目竣工投产，对经济发展的拉动作用明显增强。五年来，全社会固定资产投资由295.4亿元增加到502.3亿元，累计完成1635.7亿元，年均递增13.2%，仅2003年就新增160多亿元。五年累计完成基建投资602亿元，房地产投资249.4亿元。一些重点项目，如中铝河南分公司国债项目、宇通豪华客车生产线、登电供热机组、永通合金钢生产线、邙山干渠、郑少高速等相继竣工投产。五年新增原煤产量684万吨、发电量31亿千瓦时、氧化铝产量65万吨、汽车产量1.72万辆、污水处理能力40万吨、高速公路53.2公里、地方铁路61公里。

　　(四)市场经济体系初步建立，对外开放水平进一步提高

　　坚持在发展中推进国有企业改革。完成中央提出的国企改革和脱困“三年两大目标”，解决了一些比较突出的企业历史遗留问题。按照现代企业制度的要求，积极推进300余户国有大中型企业改革和股份制改造。

　　“郑州燃气”在港上市成功，上市公司达到8家。完成了白鸽、百文、豫联、宇通、新烟、郑烟等企业集团的改制重组。非公有制经济发展迅速，占全市经济总量的比重达到45.1%。市政府部门由49个减少到42个，事业单位初步实行了聘用制，行政审批事项由1200多项减少到317项。农村税费改革取得成效，农民负担减轻34.6%。推行部门预算，实行国库集中支付制度，公共财政体系初步建立。市场体系建设全面展开，经济秩序得到改善。社会保障体系框架基本形成。投融资体制改革深化，融资渠道不断拓宽。狠抓土地市场秩序的治理整顿，经营性用地基本实现了公开出让。户籍制度改革进展顺利。

　　投资环境显著改善，对外开放步伐加快。五年来，我们一直坚持大力整治经营环境，规范市场秩序，进一步简化了办事程序，设立了办事大厅，完善了便民措施，城市环境、政策环境、体制环境、法制环境得到明显改善。在世界银行《2003年中国投资环境报告》排序中，我市投资环境位居前列；“中国质量万里行”评价郑州整体服务质量为“优秀”。招商引资质量明显提高，在我市投资的世界500强企业已达15家。成功举办了两次“芝麻网”年会和世界客属第十八届恳亲大会，提高了郑州在国内外的知名度。累计批准设立外商投资企业371家，外商直接投资6.14亿美元。直接进出口由8亿美元增至14.1亿美元，年均增长12%。对外经济合作逐步扩大。高新技术产业开发区连续三次被评为全国先进开发区，经济技术开发区升格为国家级开发区，出口加工区正在加紧建设。对外交往日益活跃，新结国际友好城市5个。旅游环境显著改善，被评为全国首批优秀旅游城市。

　　(五)城市建设步伐加快，城市面貌明显改观

　　坚持高起点编制、高标准实施城市规划。首次采用国际招标方式，完成郑东新区起步区规划方案，各项专项规划基本完成。编制了中心城区12个方面专项规划，并严格按照规划实施。

　　按照省委、省政府提出的“三年出形象，五年成规模”的要求，强力推进郑东新区建设，近两年累计投入资金达55亿元。起步区和龙子湖区内29条道路基本竣工。中央商务区建设已安排项目35个，国际会展中心完成投资6.2亿元，内外环高层建筑有27栋开工建设。“三河一渠”治理全线开工。批准入驻高校10所，有5所已经开工。对中心城区实施综合整治，市区面貌日益净、绿、亮、美。环卫基础设施日益完善，王新庄污水处理厂已竣工投产，市区旱厕全部改造，创新了保洁机制，道路清扫、保洁水平进一步提高，国家卫生城市已通过省级验收。共建成6个公园、15个绿化广场、80个街头游园，改造背街小巷319条，对出入市口和东风渠、熊儿河进行了综合整治绿化，人均公共绿地面积由4.23平方米增加到6.8平方米，被命名为全国园林绿化先进城市和省级园林城市。基本实现了市区路灯全覆盖，夜景照明成效显著。不断加强和完善基础设施建设，水、电、气、热、通信等供应和保障能力提高。完成了郑州军用机场搬迁，建设、改造了环城快速路、紫荆山路、东西大街、金水路、中原西路等，缓解了市区交通压力。城市框架不断拉大，按新口径统计，建成区面积增加到212.4平方公里，常住人口达到322万人，全市城镇化率达到57%。

　　大力推进城市管理体制改革，长效管理机制初步形成。建立完善了“两级政府、三级管理、四级网络”新体制。推行城市管理综合执法，初步解决了多头管理、重复执罚等问题，脏乱差现象得到有效治理。公交、客运出租运营秩序明显改善。环境保护实现“一控双达标”，城市环境综合整治考核连续9年在全省排名第一。

　　(六)就业再就业工作力度加大，社会保障体系进一步完善

　　五年来，通过改善就业环境、创造就业条件、建立就业服务网络、开发就业岗位，累计培训下岗失业人员13.2万人，27.2万人实现就业和再就业，城镇登记失业率控制在3.5%以内。

　　社会保障体系初步建立，保障网络逐步完善。认真落实“两个确保”，养老金和下岗职工基本生活保障金发放率均保持100%。建立健全了退休人员社会化服务网络，16.6万名企业离退休人员实现了社会化管理。失业、工伤、医疗保险顺利推进。城市居民最低生活保障实现了应保尽保，在就业、住房、医疗、教育等方面出台了低保配套政策。在全省率先实行了农村特困家庭救助制度。农村新型合作医疗试点进展顺利。

　　(七)民主与法制建设得到加强，社会秩序安全稳定

　　自觉接受人民代表大会及其常委会监督，接受人民政协的民主监督，认真听取民主党派、工商联、无党派人士、各人民团体的意见。初步建立了公众参与、专家论证和政府决策相结合的决策机制，提高了决策的民主化、科学化水平。五年共办复人大代表议案、建议和政协委员提案3467件。坚持依法治市，五年来共制定地方性法规36件、政府规章50件。廉政建设进一步加强。推进村民自治，政务、村务公开逐步规范化、制度化。加强社区建设，组织实施了“三个一”工程。社区居委会换届直选比例在全国领先。普法教育逐步深入，公民法律意识进一步提高。

　　加强社会治安综合治理，依法打击各种犯罪活动，社会治安保持稳定。与“法轮功”邪教组织斗争取得胜利。成功侦破抢劫银行等一系列大案要案，严厉打击了带有黑社会性质的犯罪活动。成功化解了郑州合行、农村合作基金会等金融风险，果断处理了一批非法集资案件。狠抓安全生产整治，安全生产形势整体趋向好转。认真搞好人民内部矛盾纠纷排查调处工作，解决群众反映强烈的热点问题，确保了省会政治稳定和社会安定。

　　(八)各项社会事业全面进步，人民生活质量明显改善

　　五年来，积极实施“2662”工业技术创新计划和“5212”农业技术创新计划，科技进步对国民经济增长的贡献率由42%提高到48.3%。实施素质教育，基础教育质量不断提高，高中教育规模扩大，高等教育面向社会大众发展。办学体制改革稳步推进，民办教育发展迅速。文化事业进一步繁荣，群众性文化活动丰富多彩，文化市场管理和扫黄打非斗争受到上级表彰。医疗卫生体制改革不断深化，医疗基础设施进一步改善，突发公共卫生事件疾病控制与救治能力提高。特别是去年，全市上下众志成城，取得了抗击非典的阶段性重大胜利，实现了“不发生一例非典确诊病例、不漏治一名非典疑似患者、不使一名医护人员倒下”的目标。市体育代表团在省以上运动会上取得优异成绩，群众性体育活动蓬勃开展。市科技馆、博物馆、艺术宫、杂技馆、航海体育场等一批文化、体育设施工程竣工投入使用。人口和计划生育各项工作稳步推进，人口出生率下降2.49个千分点。国防教育、民兵、征兵、优抚安置工作顺利开展，军政军民关系更加密切，连续四次荣获“全国双拥模范城”称号。

　　全市广大干部群众深入学习邓小平理论和“三个代表”重要思想，深入开展“三讲”教育活动，普遍进行了社会主义道德教育、民主法制教育和纪律教育。先后抽调7331名机关干部，分三批下乡开展驻村工作。精神文明建设取得新进展。在创建活动中荣获“全国文明城市创建工作先进单位”称号。

　　我们始终把逐步提高人民生活水平作为政府工作的出发点和落脚点，努力提高城乡人民收入，改善生活质量。市区城镇居民人均可支配收入由5721元增加到8647元，年均增长9.7%；农民人均纯收入由2530元增加到3631元，年均增长7.5%。城市居民人均住房面积达到22.4平方米，五年增长31%；农民人均住房面积39.7平方米，增长49.8%。围绕群众生活，连续5年向社会承诺办好十件实事，坚持实行60岁以上老人免费乘坐公交车，公园和公厕免费对公众开放。人民生活水平显著提高。

　　新闻出版、广播电视、人事、统计、审计、物价、侨务、对台事务、民族宗教、社会科学、地方志、档案管理、气象、人防等工作都取得了新的成绩。

　　各位代表，过去的五年，是我市经济实力增长较快的五年，是城市面貌变化较大的五年，是人民群众得到实惠较多的五年。这些成绩的取得，是省委、省政府和市委正确领导的结果，是全市人民共同奋斗的结果，是市人大、政协监督支的结果，是驻郑人民解放军、武警官兵、中央驻郑单位和省直厅局、各民主党派、工商联、各人民团体、离退休老同志和海内外各界人士共同努力、鼎力支持的结果。在此，我代表市人民政府，向给予政府工作大力支持的人大代表和政协委员，向全市广大工人、农民、知识分子、干部、离退休老同志、驻郑人民解放军、武警官兵、公安干警、各民主党派、工商联、人民团体以及各界人士，表示崇高的敬意！向关心和支持郑州建设的港澳台同胞、海外侨胞、国际友人，表示衷心的感谢！

　　五年来，政府的各项工作虽然取得显著成绩，但还存在不少困难和问题，群众还有一些不满意的地方：一是经济实力和辐射带动能力不强，在全省的首位度不高，与省会城市地位不相称。二是产业结构不尽合理，支柱产业缺乏规模优势，总量偏小，在全国知名的大企业大集团太少。三是对外开放的体制、机制创新任务还很艰巨，利用外资规模过小，经济外向度较低，不适应经济全球化和日益激烈的城市竞争。四是农民增收缓慢，城乡不少低收入居民生活还比较困难。五是就业岗位不能充分满足就业需求，就业压力仍然很大。六是资源保护、环境治理与生态建设任务繁重，城市管理的长效机制有待巩固和完善。七是一些严重刑事犯罪案件屡有发生，重大安全事件接连出现，给人民群众生命财产造成严重损失。八是群众关注的一些热点难点问题没有得到及时有效解决。九是部分工作人员存在形式主义和官僚主义作风，政府自身建设和反腐败任务还很重。所有这些，都需要我们在今后的工作中切实加以改进和解决。

　　二、今后五年的目标任务

　　各位代表，今后五年我们面临着不可多得的发展机遇。从国际环境看，和平与发展依然是当今时代两大主题，世界经济形势趋向好转，国际产业转移加快，有利于我们进一步扩大对外开放和加快产业结构调整。从国内看，全面建设小康社会的热潮已经兴起，举国上下百舸争流，周边城市竞相发展，沿海地区产业和资本向内地转移，有利于促进我市消费结构和产业结构的升级。特别是最近国家提出了“促进中部地区崛起”的战略，省委、省政府要求郑州提高首位度，增强对中原城市群的带动作用，为我市加快发展提供了契机。同时，我市生产总值已突破千亿大关，综合经济实力和发展后劲明显增强，具备了发展全面提速、再上新台阶的基础和条件。但是，我们还应清醒地看到，当今世界局势还有许多不确定因素，在国内新一轮发展热潮中，全国大中城市，特别是周边城市发展势头迅猛，形势逼人，时不我待。我们要树立强烈的忧患意识，抢抓机遇，迎难而上，以更大的气魄和胆略，推进建设大郑州、建设国家区域性中心城市，无愧地担负起中原城市群经济隆起带核心城市的重任。

　　今后五年政府工作的总体要求是：以邓小平理论和“三个代表”重要思想为指导，全面贯彻党的十六大和十六届三中全会精神，坚持以人为本的科学发展观，不断深化改革，扩大开放，加快先进制造业基地、现代服务业中心、现代农业示范区建设，加强民主法制建设和精神文明建设，创建生态郑州、平安郑州、信用郑州，促进经济社会全面协调可持续发展，为实现中原崛起和全面建设小康社会目标奠定坚实的基础。

　　主要预期发展目标是：

　　综合实力再上新台阶。生产总值保持年均13%左右的增长速度，到2008年突破2000亿元大关；人均生产总值达到3000美元；地方财政收入达到180亿元。

　　经济结构进一步优化。到2008年，三次产业的比例预期为3.0 51.1 45.9，工业、农业产业结构进一步优化，非公有制经济比例达到60%以上，城乡经济协调发展，现代服务业水平有大幅度提高。

　　城市建设和管理再上新台阶。到2008年，建成区面积力争达到255平方公里；全市城镇化率达到62%以上；管理水平进一步提高，创建国家卫生城市、园林城市、环保模范城市和文明城市，建成国内最适合创业发展和生活居住的城市之一。

　　开放带动更具新活力。经济管理体制逐步与国际规则接轨，利用外资规模进一步扩大，对外开放提高到一个新水平。

　　市场经济体制更加完善。经济发展环境明显改善，民营经济迅速壮大；国有资产管理体制进一步理顺，现代企业制度基本建立；农村改革进一步深化，农村经济体制逐步完善；统一、开放、竞争、有序的市场体系基本形成，市场经济秩序不断好转。政府的经济调节、市场监督、社会管理和公共服务职能显著增强。

　　各项社会事业全面发展。科技、教育、文化、卫生、体育、计划生育等各项社会事业进一步发展，人口健康水平大幅度提高，市民多元化的物质和精神需求逐步得到满足。

　　人民生活水平明显提升。社会保障体系日益健全，就业问题得到较好解决，生活质量进一步改善。城镇居民人均可支配收入和农民人均纯收入年均增长8%以上。

　　依法治市水平进一步提高。民主法制建设更加完善，政府依法行政水平明显提高，市民法律意识显著增强，建设“平安郑州”，实现社会安全稳定、治安秩序良好、人民安居乐业。

　　精神文明建设取得新进展。思想道德教育深入人心，城市意识、省会意识不断增强，艰苦创业、团结奋进、敢为人先的精神得到弘扬，社会信用体系建设逐步完善。

　　完成上述目标任务，必须坚持以下几点：

　　一是坚持解放思想，实事求是，与时俱进。要从国际化和城市竞争的大格局中，探求建设现代化大郑州的新路子。立足创大业、干大事、干实事，建设大郑州；按照“发展要有新思路，改革要有新突破，开放要有新局面，各项工作要有新举措”的要求，依靠全市人民的智慧和力量，把省会现代化建设不断推向前进。

　　二是坚持把发展作为第一要务，聚精会神搞建设，一心一意谋发展。坚持在发展中解决前进道路上的困难和问题，不断壮大经济实力，增创发展新优势。紧紧围绕发展生产力这一中心，用发展的思路和办法，积极应对各种挑战，破解各种矛盾和问题，特别是长期困扰我市发展的城市建设资金不足、工业发展滞后、就业形势严峻、城乡二元化矛盾突出等问题。

　　三是坚持深化改革，扩大对外开放。坚持依靠体制创新，完善市场经济体制，依靠科技创新，推动经济结构调整，依靠对外开放，拓展发展空间，努力提高经济运行质量。在改革开放中，勇于探索，开拓创新，加快产业结构优化升级，促进传统产业、高新技术产业和服务业协调发展。按照开放型、现代化大郑州的建设标准，搞好城市规划建设管理，提高综合竞争力。

　　四是坚持以人为本、执政为民，切实为人民群众办实事、办好事。努力践行全心全意为人民服务的宗旨，维护和发展人民群众的根本利益，把执政为民、求真务实体现在具体工作中，千方百计解决人民群众普遍关注的热点和难点问题，在不断发展经济的基础上，不断提高人民生活水平，改善生活质量。

　　五是坚持科学的发展观，正确处理各方面矛盾和关系。按照十六届三中全会统筹发展的精神，正确处理改革发展与稳定的关系，城乡经济协调发展的关系，工业经济和商贸经济发展的关系，老城区保护改造和新区开发建设的关系，物质文明、政治文明和精神文明建设的关系，人口、资源、环境之间的关系，实现郑州经济社会全面、协调、可持续发展。

　　三、2004年的工作建议

　　2004年是我市实施全面建设小康社会规划的开局之年，做好今年的工作，对于保持经济社会发展的良好势头，具有十分重要的意义。主要预期目标是：生产总值增长13%左右；全社会固定资产投资增长35%；社会消费品零售总额增长11%；地方财政收入增长14.5%；外贸出口增长12%，实际利用外资增长30%左右；居民消费价格总水平上涨2%左右；城镇居民人均可支配收入增长8%，农民人均纯收入增长7%；人口自然增长率6‰；城镇登记失业率3.5%。同时，要努力实现以下工作目标：新增就业再就业10万人；工业技改投资100亿元；新增非公有制经济增加值100亿元；地方财政收入100亿元；新设立外资企业100家；规模以上工业销售收入1000亿元。

　　为确保完成上述目标任务，应重点抓好以下工作：

　　(一)壮大工业经济规模，加快工业化进程

　　围绕把郑州建成先进制造业基地的目标，进一步壮大工业经济规模，做强做大支柱产业，重点抓好100家工业企业增收增效。以中铝河南分公司、郑煤、郑铝、登电、豫联等企业为主体，加快大型铝工业项目和能源项目建设，拉长煤电铝产业链条，促进铝产品结构升级。以宇通、日产、少林、红宇等企业为主体，促进汽车工业升级换代、做强做大。加大技术引进、合资合作力度，提高汽车工业零部件配套能力。加快新烟、郑烟集团和三全、思念等龙头企业的发展，尽快建成国内重要的食品生产加工基地。以郑纺机、郑煤机等优势企业为主导，整合装备制造业。以开发高附加值产品为方向，对纺织工业实施调整升级，加快纺纱、织布、印染、服装生产基地建设，拉长纺织工业链条。

　　积极培育以电子信息、生物工程和制药、新材料等为代表的高新技术产业，争取尽快形成新的支柱产业。依托国家863软件孵化基地、安飞集团，建设芯片、软件等一批电子信息产业项目；依托省生物医药产业孵化基地，扶持竹林众生、新郑制药等企业，加快制药、生物工程、基因治疗等关键技术的研发；依托省超硬材料基地，扶持华晶超硬材料产业园，加快新材料企业上规模。

　　狠抓工业投入，实施名牌战略，培育拳头产品，推进产业结构优化升级。抓好100项重点工业技改项目，积极争取国家和省技改资金，加快我市传统工业的技术改造，推进新技术、新工艺的开发和应用，提高工业经济运行质量。

　　加快集群经济和区域经济发展，引大型项目和高科技企业向园区集中。加快高新区技术创新和高新技术产业化基地建设，以及经济技术开发区外向型经济基地建设，努力把出口加工区建成全省重要的出口加工基地。努力打造郑汴洛郑州段工业走廊，发展各县市区有优势特色的工业群区，促进县域经济发展。

　　搞好重点企业跟踪服务，推进资源整合。继续实行领导联系重点企业制度，对重点企业和增量大户在资金、技术、能源、交通等方面给予大力支持，坚持向重点企业派驻联络员制度，及时解决企业发展中遇到的困难和问题。加强安全生产工作，加快应急救援体系建设，遏制各类特别是重特大生产安全事故发生。

　　(二)推动新兴服务业发展，提升服务业现代化水平

　　加快现代化商贸城建设。发挥现有商业资源优势，加大整合力度，积极发展连锁经营、物流配送、电子商务等新型业态。在引进大型商贸企业的同时，积极利用外资嫁接改造国有商业企业。加快大上海城、家世界购物广场等在建项目的建设进度，积极做好沃尔玛等项目建设前期工作。进一步整合商品交易市场，加快15个交易园区建设。大力推进现代物流业发展，加快圃田物流园区建设，进一步做好郑州现代物流中心等项目建设过程中的协调工作；培育豫新、长通、思达、邮政等物流配送中心，逐步形成一批具有现代化经营能力的物流企业。继续抓好会展中心建设，搞好郑交会等会展活动，繁荣会展经济。

　　整合旅游资源，组建旅游集团。发挥财政性资金的引导作用，吸引社会资金投资旅游景区建设。抓好嵩山、黄河、轩辕故里等风景区特别是少林景区的建设、改造。办好旅游节庆活动，形成一批具有中原文化特色的旅游观光项目，提升我市旅游业整体形象和水平。

　　搞好房产开发和经济适用房建设。进一步放宽公房出售政策。继续推进和落实住房货币化补贴政策。加强和规范住房公积金、维修基金管理。搞活住房二级市场。规范物业管理，优化和改善居住环境。

　　积极发展信托、票据、拆借、证券、期货等金融市场；创造条件，吸引国外金融投资机构，参与金融企业重组。完善金融服务体系，鼓励发展金融担保业和信息、会计、法律、咨询等中介服务业，努力把郑州建成中西部地区重要的金融服务中心城市。

　　(三)切实解决“三农问题”，坚持农业基础地位不动摇

　　切实把解决好农业、农村、农民问题作为全部工作的重中之重。按照建设都市型、近郊型和远郊型农业圈层的思路进行区域布局调整。大力发展优质、高产、高效、生态、安全农业，提高农产品质量和竞争力。积极发展绿色名特优新农副产品和品牌农业，提高农业的比较效益。推进农业产业化经营，力争建成全省一流的现代农业示范区。继续搞好绿化工程建设，完成造林28万亩。发展畜牧业规模养殖，加快奶牛养殖小区和黄河滩区绿色奶牛养殖带建设步伐。发展优质、高效水产业。逐步扩大农产品市场准入范围，让群众吃上“放心菜”、“放心肉”。大力发展农产品加工业，积极发展农产品批发市场和农民专业合作经济组织，完善农产品市场信息服务体系建设。

　　坚持“多予、少取、放活”的方针，加快农村经济发展，促进农民增收。完善开发式扶贫机制，加快移民脱贫步伐。加大扶贫投入力度，市财政安排1000万元用于扶贫开发。全市所有贫困村免征农业税，全市农业税税率平均降低3个百分点，五年内逐步取消农业税。稳步推进农用地承包经营权流转，逐步发展规模经营。实行严格的耕地保护制度，依法加强耕地管理，完善土地征用办法和补偿机制。实行农民求职登记制度，加强职业技能培训，加快农村剩余劳动力的转移。加大农民工工资清欠力度，努力保障按时足额支付。落实种粮直接补贴政策。加强农资价格监管，严厉打击坑农、害农等行为。

　　进一步加大农业投入力度，加强农业和农村基础设施建设。搞好节水灌溉等“六小工程”和农田水利建设，新增灌溉面积2万亩。推广应用先进实用农机具。搞好农村水电路等基础设施建设，改善农民生产生活条件。

　　(四)抓好重点项目建设，继续保持良好的投资势头

　　继续坚持投资拉动，抓好100项重点项目工程。开工建设南三环联接线、郑少高速与航海路立交、中电燃气调峰电厂2×30万千瓦供电、新力电力2×20万千瓦供热、赵家寨煤矿、中美铝业30万吨氧化铝、澳柯玛物流一期、家乐福超市、郑州一中迁建、五院和颐和医院工程等57个项目。加快绕城高速、农业路铁路立交、登电、豫联、五龙口污水处理厂等29个在建项目进度，107辅道建成通车。郑花路综合整治、华润发电机组、47中迁建等16个项目年内完工。做好中电产业园、香江现代物流商贸园区等14个项目的前期工作，并尽快开工建设。

　　对重点项目实行领导联系制度，及时协调解决项目建设中遇到的困难和问题，确保按进度顺利推进。实行严格的质量管理制度，全面落实项目法人责任制、招投标制、建设监理制、竣工验收制、工程质量终身负责制。

　　(五)进一步加快城市化进程，促进城乡协调发展

　　以中央商务区为重点，进一步加快郑东新区建设。第一、二批进入中央商务区的高层建筑基本竣工，第三批进入的8个住宅楼和国际会展中心主体工程基本完成；省艺术中心主体框架基本形成；会展宾馆和商业步行街争取年内开工建设。认真搞好管网和道路系统建设。加快龙子湖高校园区和物流园区建设进度。着手森林公园和生态功能区建设。按规划搞好中央商务区景观绿化。力争实现“三年出形象”的预期目标。

　　按照“一年一小变，三年一大变”的建设要求，继续加大中心城区保护改造力度。完善市区路网结构，新修和打通二环路、文化北路、沙口路等主次干道，开工建设农业路与花园路立交及经三路、城东路与金水路立交等工程，改造部分支路和背街小巷。继续抓好道路街景整治，建成一批街景整治示范街、精品街，行道树景观和夜景照明示范街。抓好熊耳河、金水河、东风渠和出入市口及铁路沿线等的绿化整治。重点改造关虎屯、燕庄等18个城中村。完善市区公共服务设施建设。进一步加强清扫保洁工作。力争年内基本达到国家卫生城市、园林城市的标准要求。

　　加快五市一县基础设施和环境建设，高品位、高起点建设20个重点镇，搞好乡村规划，实现中心城区、卫星城、小城镇在规划上衔接、功能上联接、产业上对接。

　　完善“两级政府、三级管理、四级网络”城市管理体制，建管并重，进一步提高城市管理水平。规范国土资源管理，清理闲置国有土地，整治停缓建工程。加强水资源管理，搞好城市节水。优化交通安全管理，确保市区道路畅通。以创建环保模范城市为载体，加大环境综合整治力度，推进垃圾“三化”处理，推广乙醇汽油，为市民创造舒适、安全、优美的生活居住环境。

　　坚决维护城市规划的严肃性，各项建设必须符合总体规划要求。市、区都要发挥规划的调控作用，严格按详细规划实施。充分发挥省会规划委员会的作用，确保省会规划的集中统一管理。

　　(六)继续深化各项改革，加快非公有制经济发展

　　进一步加大国有企业改革力度，实行一企一策，基本完成工业、商业以及其他国有及国有控股企业改革任务。推进已改制企业在完善法人治理结构、促进资产多元化、建立内部“三项制度”等方面深化提高。加快投融资体制改革，支持新组建的建投、路桥、地产等政府投资主体按照市场机制运作。进一步推进城市公用事业改革，开放市政公用行业市场。不断推进行政审批制度改革，完善办事大厅服务功能。巩固农村税费改革成果。深化财政国库管理和财政预算管理制度改革。完善各项配套措施，解决户籍改革中遇到的问题。深化政府机构和事业单位人事制度改革。

　　大力发展和积极引导非公有制经济。进一步清理和修订不利于非公有制经济发展的法规和政策，放宽市场准入条件，鼓励民营企业投资基础产业和基础设施建设。重点抓好100家非公有制企业，支持科技型、外向型、农业型、扶贫型和劳动密集型民营企业发展。加强中小企业创业服务体系建设，引导民营企业技术创新和制度创新，走规模化、集约化道路。

　　(七)进一步扩大对外开放，大力发展开放型经济

　　加大招商引资工作力度，力争利用外资有新的突破。支持引导外资参与企业改革，放宽外商投资领域，吸引外资参与社会事业和旧城改造。完善招商引资项目库，加强项目推介，抓好项目跟踪落实。组织参加厦洽会、高交会等经贸活动，利用举办首届世界传统武术节的机遇，发挥郑东新区、高新区、经济开发区和出口加工区对外开放的主窗口作用，扩大招商引资，实现新设立外商投资企业100家的目标。

　　进一步扩大对外贸易和经济技术合作。加强出口企业队伍建设，力争全市拥有进出口经营权的企业达到1000家。大力拓展欧美市场，开拓俄罗斯、非洲、南美等新兴市场。鼓励支持企业走出去承包工程、投资办厂，带动技术设备和劳务出口。

　　继续实施“东引西进”战略，积极开拓国内市场，扩大对内开放。加强与上海、广东、香港及江浙等城市和地区的经贸合作。积极参与西部大开发，实现产业和市场的拓展。

　　(八)做好财税金融工作，充分发挥财政、金融对经济社会发展的支持作用

　　抓住中央继续实施积极的财政政策的机遇，尽可能多地争取国债资金，支持经济发展，重点加大对我市农业和农村基础设施的投入力度。加大科技投入，支持科技研发和高新技术产业化。积极支持非公有制经济发展，营造公平竞争的财税环境。坚持依法治税，强化税收征管，努力使财政收入规模与经济发展相适应。加强支出管理，强化监督，提高财政资金使用效益。今年省与市、市与区财政体制进行了调整，要认真搞好衔接，确保平运行。

　　发挥金融对拉动投资和消费的支持作用。加强银企沟通与合作，争取金融机构不断增加在我市的贷款投放。发挥中小企业信用担保机构作用，解决中小企业贷款难问题。规范发展各类消费信贷业务，增强对消费需求的信贷支持。争取和支持国内外各类商业银行、证券公司、保险公司在我市组建法人机构或分支机构。扩大上市公司规模，做好上市公司资产重组和再融资工作。

　　(九)切实做好就业和社会保障工作，提高人民生活水平

　　进一步落实再就业的各项政策，完善各项服务措施。重视发展劳动密集型产业，扶持中小企业、民营经济，大力开发公益性就业岗位，用足用好促进再就业的各项政策和资金，引导下岗失业人员通过自谋职业、自主创业和合伙经营实现再就业，确保全年新增10万人就业再就业目标的实现。切实做好大中专毕业生的就业指导和服务工作，为高校毕业生自主创业提供政策支持。

　　继续做好“两个确保”和城市“低保”工作，切实落实“三条保障线”。进一步加大社会保险扩面征缴力度，确保基金征缴率达到90%以上，确保养老金足额和社会化发放率100%。加大财政资金投入力度，完善城乡社保和社会救济体系，做到城市“低保”资金足额、及时到位，符合条件的农村特困家庭全部纳入救助范围。稳步扩大医疗保险、工伤保险覆盖面，认真做好国有破产企业、困难企业退休人员和农村特困户社会医疗救助工作。进一步完善廉租房制度，努力满足“双困”家庭住房需求。

　　(十)坚持依法治市，努力建设平安郑州

　　加大依法治市力度，加强治安防控体系建设，争创“全国社会治安综合治理工作先进市”。严格落实社区一警一室，进一步提高快速反应能力。广泛开展警民联防、军警联防、企地联防和安全院、楼、村活动，构筑群防群治体系。加大对都市村庄综合治理力度，加强对流动人口的管理和服务工作。坚决扫除“黄赌毒”等社会丑恶现象，创建无毒社区、无毒乡村。保持严打高压态势，严厉打击各种刑事犯罪。高度重视人民群众来信来访，解决好涉法信访问题。强化不稳定因素排查调处责任制，完善矛盾纠纷调处体系，积极预防、妥善处置突发性群体事件。依法惩治“法轮功”等邪教组织。加大反恐怖斗争力度。完善基层司法所和法律服务网络建设，推进司法公正，提高司法效率，树立司法权威，营造长期和谐的社会环境和公正高效的法制环境。

　　(十一)坚持科教兴郑战略，加快文化卫生体育等各项社会事业发展

　　进一步加大科技投入，大力推进科技进步与创新。抓好制造业信息化、农村信息化和科学数据资源共享三大工程建设，加快实施“2662”工业技术创新计划和“5212”农业技术创新计划，促进和提升产业竞争力。加强人才资源开发，加强与科研院所和高校的合作，支持省大学科技园建设，加快科技成果转化和产业化。力争今年科技进步对经济增长的贡献率达到50%。

　　加强基础教育、职业教育、素质教育和爱国主义教育。加快发展优质基础教育，完善市区中小学的布局规划，加快建设4所高中，70%以上乡镇建成中心幼儿园。整合中等职业教育资源，扩大规模，招生达到2.5万人。推进市属高校布局和学科专业调整，各类高校在校生达到5.5万人。完善农村教师工资保障机制。

　　大力发展社会主义先进文化。实施文化精品工程，进一步繁荣文艺创作，以文化艺术的形式宣传郑州，推介郑州。深化文化新闻产业改革，建好郑州歌舞剧院。加强文化市场管理，继续开展扫黄打非。加强商城遗址、文庙、城隍庙等文物的保护、管理和利用工作。大力开展读书活动，建设学习型城市。

　　进一步加强公共卫生基础设施建设，提高应对突发性公共卫生事件的应急能力。加强全市疾病预防控制、突发公共卫生事件医疗救治、重大疫情信息网络三个体系建设，尽快建立郑州市紧急救援指挥中心。加强乡镇卫生院建设，完善农村三级预防保健网络和新型合作医疗制度。继续做好社区卫生服务工作。

　　广泛开展全民健身活动，不断提高体育竞技水平。抓好第九届全国中运会6个场馆建设和各项筹备工作。

　　高度重视人口和计划生育工作。坚持一把手亲自抓、负总责，切实做到责任、措施、投入到位，人口自然增长率控制在6‰以内。建立完善综合治理、利益导向和养老保障机制，对满60岁的农村独生子女父母每年给予600元补助。切实解决二孩率偏高、流动人口管理薄弱等突出问题。加强老龄工作，认真解决离退休人员生活、就医问题。

　　加强国防教育，做好复转军人和随军家属安置工作，落实军休干部待遇，深入开展双拥活动，再创全国“双拥”模范城。继续做好统计、审计、民族、宗教、民政、民兵等各项工作。

　　各位代表，办好直接关系群众生活的实事，是市政府解决人民群众实际问题的有效途径。今年，通过征求社会各界意见，确定的10件实事是：一、新增城镇就业岗位10万个，推进农民求职登记服务工作。二、城市居民最低生活保障标准由180元提高到200元，符合低保条件的城市低保对象应保尽保，符合条件的农村特困户全部实行救助。三、加大中心城区保护改造投资，重点对断头路、积水点、街头游园等基础设施进行改造，开工建设西流湖公园。四、净化市区大气环境，新增燃气用户2.3万户，空气质量二级以上天数达到65%以上。五、西部缺水山区建设集雨节灌水窖1万个。六、改造、新建农村公路100公里。七、完成中小学危房改造10万平方米。八、建设郑州市医疗紧急救援指挥中心，6县(市)、上街区建立分中心，每个乡镇卫生院设立急诊科，形成覆盖全市的医疗紧急求救网络体系。九、向社会提供经济适用房40万平方米；“双困”家庭实行廉租住房制度。十、完成沿黄风沙源生态治理造林10万亩。上述10件实事，我们将明确责任，保证投入，确保年底实现。

　　四、努力建设服务型政府

　　全面完成今后五年政府工作的各项目标任务，必须坚持依法行政，不断推进服务型政府建设，全面提高行政效率和管理水平。

　　(一)全面推行依法行政，提高依法行政水平

　　要按照法定权限和程序行使权力、履行职责，依法处理经济、社会事务。贯彻执行《中华人民共和国行政许可法》，抓紧清理与之相悖的规章和规范性文件。深化行政审批制度改革。进一步推进相对集中行政处罚权工作，确保行政执法的合法、公正、文明、规范。进一步强化行政监督和行政复议工作，建立和完善行政机关及其工作人员行政责任追究制度。

　　(二)加快行政管理体制改革，切实转变政府职能

　　按照国家和省统一部署，搞好新一轮政府机构改革，严格控制机构、编制和人员。进一步转变政府经济管理职能，注重运用经济和法律手段管理经济，真正把政府经济管理职能转到主要为市场主体服务和创造良好的发展环境上来。提高服务效率，建立便民服务机制。创新政府管理方式，推进电子政务建设，加大信息资源开发力度，优化行政流程，全面提高行政运转效能。继续整顿规范市场经济秩序，严厉打击各种经济犯罪。政府带头加强诚信体系建设，建设诚信郑州，建立以道德为支撑、产权为基础、法律为保障的社会信用制度，营造良好的发展环境。

　　(三)完善科学民主决策机制，推进民主政治建设

　　各级政府要坚决贯彻人大及其常委会决议，坚持重大事项报告制度，自觉接受人大及其常委会监督。认真接受政协的民主监督，广泛听取各民主党派、工商联、无党派人士、社会团体的意见和建议，把政治协商纳入政府决策程序，为民主监督和参政议政创造良好条件。完善科学民主决策机制，推行政务公开，建立健全决策论证、专家咨询、社会听证、社情民意反映、人民建议征集等制度。对重大决策，要进行反复比较论证，确保决策的科学性。

　　(四)弘扬求真务实精神，切实转变政府工作作风

　　加强公务员队伍建设，全面提高公务员素质。政府全体工作人员特别是各级领导干部，要树立正确的政绩观，不搞脱离实际、劳民伤财的“形象工程”和“政绩工程”。按照执政为民的根本要求，强化群众观念，弘扬求真务实精神，扑下身子，真抓实干，做好涉及人民群众利益的每一项工作，多为群众办实事、办好事，真正做到“权为民所用，情为民所系，利为民所谋”。

　　坚持从严治政，推进勤政廉政建设。加大行政监察和政风、行风整顿力度，继续搞好为纳税人服务和社会各界民主评议活动，切实纠正部门和行业不正之风。努力建设一支廉洁奉公、勤政为民、业务精通、作风优良的政府公务员队伍。

　　各位代表，郑州是充满生机和活力的城市，展望未来，郑州的明天将更加美好。让我们紧密团结在以胡锦涛为总书记的党中央周围，高举邓小平理论和“三个代表”重要思想伟大旗帜，认真贯彻党的十六大和十六届三中全会精神，在省委、省政府和市委的领导下，紧紧依靠全市人民，求真务实，团结奋进，抓住机遇，加快发展，为实现中原崛起和全面建设小康社会的宏伟目标而努力奋斗！
